# Combined Conventional–Digital Workflow to Fabricate a Palatogram for Speech Rehabilitation in a Single Arch Completely Edentulous Class II Patient With Dysarthria: A Clinical Report

**DOI:** 10.1155/crid/2036019

**Published:** 2025-03-21

**Authors:** Abdulaziz M. Alqarni, Walaa A. Babeer, Thamer Y. Marghalani

**Affiliations:** Department of Oral and Maxillofacial Prosthodontics, Faculty of Dentistry, King Abdulaziz University, Jeddah, Saudi Arabia

**Keywords:** complete, denture, dysarthria, palate, software, speech, tongue

## Abstract

This clinical report describes the creation of a palatogram for speech improvement in a single-arch, completely edentulous, skeletal Class II patient with dysarthria. The treatment focused on occlusal considerations specific to Class II denture wearers and capturing the tongue–palate contact regions during speech. The outcome was assessed by consulting a speech therapist using speech analysis software.

## 1. Introduction

Loss of teeth and supporting structures not only reduces masticatory efficiency but also disrupts the speech articulatory mechanism, leading to speech disorders from altered speech patterns and significantly reducing speech intelligibility [1, 2]. Consequently, affected individuals experience social and emotional difficulties due to communication difficulties, significantly impacting relationships and functionality [3]. Dysarthria, characterized as “a neurologic motor speech impairment causing the speech musculature to be slow, weak, and/or imprecise,” adversely affects breathing, voice production, resonance, and oral articulation [4, 5]. Moreover, communication becomes difficult for individuals with dysarthria due to their abnormal-sounding speech and decreased clarity [6]. Complete tooth loss, particularly in edentulous patients with dysarthria, further complicates the speech mechanism and treatment [2]. Notably, Pound suggests distinct sound formation between patients with Class II and Class I malocclusion [7], where the co-occurrence of dysarthria in the former presents a complex clinical scenario.

Phonetic considerations are crucial when creating complete dentures since the mouth is essential in phonation and significantly affects communication [2, 8]. This requires sufficient background knowledge of phonation principles among prosthodontists who create dentures for the restoration or improvement of normal speech.

Static articulators (teeth and hard palate) and dynamic articulators (lips and tongue) contribute to amplifying and modifying sound. During speech, the tongue specifically interacts with the hard palate, alveolar ridge, and teeth to articulate various sounds [9]. Thus, the occurrence of the type of tongue–palate contact is evaluated using palatograms [10], which are “a graphic representation of the area of contact between the tongue and palate during speech” [11]. Studies have suggested that palatograms can aid in restoring natural palatal contours, potentially reducing speech adaptation time [12, 13]. However, to the best of our knowledge, palatograms have never been reported to manage maxillary complete dentures in Class II patients with dysarthria. This case report is aimed at utilizing a palatogram to manage and fabricate maxillary complete dentures in Class II patients with dysarthria.

## 2. Clinical Report

**Figure 1:**
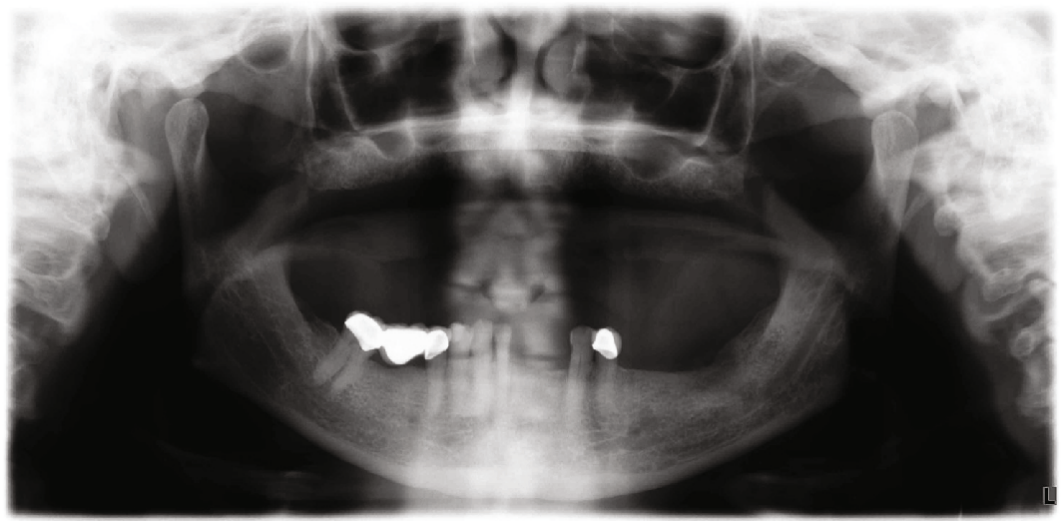
Panoramic radiograph.

The patient provided written informed consent for photographic documentation and case publication. He was a 67-year-old male who visited the prosthodontic office for the replacement of his old dentures due to staining, wear, and damaged acrylic teeth. His medical history revealed hypertension and a previous stroke 3 years ago, resulting in his dysarthria. His current medications include aspirin, amlodipine, clopidogrel, and Lipitor. According to the prosthodontic diagnostic index (PDI) classification, the patient had a Class IV edentulous maxilla requiring occlusal vertical dimension (OVD) restoration. Moreover, the mandibular arch was classified as Class II Modification I, according to the Kennedy classification, and clinical classification revealed Skeletal Class II malocclusion with unilateral crossbite and lower anterior teeth supraeruption, as confirmed with cephalometric radiography and orthodontist consultation. The remaining teeth presented with cavities, defective restorations, and generalized Stage I Grade A periodontitis, which was characterized by a mean probing depth < 4 mm (Figure 1).

Given these findings, the patient required OVD restoration, speech therapy, and management of Class II malocclusion. Multiple treatment options were proposed in this case. One option was the use of implant-supported fixed prostheses, overdentures, or conventional complete dentures for the edentulous maxilla. The partially edentulous mandible would then be supported by fixed prostheses, replacing the teeth with the right mandibular central incisor, left mandibular central incisor, and lateral incisors, and separate single crowns for all remaining teeth. The second option was to employ a Class II Modification I, creating a unilateral removable partial denture (RPD) with the surveyed crowns. The last option was a Class II Modification II RPD with the surveyed crowns. The patient chose the option in which the maxilla was restored with a conventional complete denture and opposed by a Class II RPD, five units of fixed dental prostheses (FDPs) from the right mandibular canine to the left mandibular canine, and single crowns on the right and left mandibular first premolars.

### 2.1. Procedure

**Figure 2:**
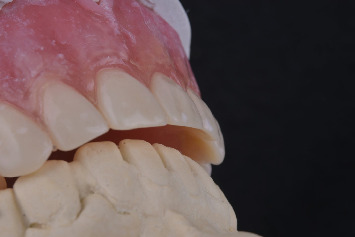
Uneven overjet during try-in.

Standard procedures were followed for creating conventional complete dentures. During the try-in step of the complete denture, as opposed to transitional RPD and interim FDP (Figure 2), an uneven and large horizontal overlap was observed due to the patient's Class II malocclusion. Additionally, he exhibited difficulty in producing clear speech, particularly with the S and Z sounds. Treatment of such patients was considered complex due to the combination of edentulous maxilla, Class II malocclusion, and dysarthria. This case brings to question whether palatograms can be used to improve speech outcomes in such cases.

**Figure 3:**
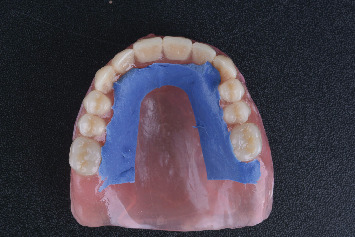
Palatogram impression.

Medium-body polyvinyl siloxane (PVS, Zhermack SpA) was applied to the palatal surface of the transitional complete denture (Figure 3). The patient was then instructed to vocalize phrases containing sounds produced by tongue–palate contact, as recommended by Goodacre et al. [10]. It should be noted that in this case, the sentences were modified to Arabic, as illustrated in Table 1. Subsequently, the excess midpalatal impression material was removed.

A reference denture (palatogram denture) and a trial denture were used. After obtaining a wash impression, the external surface of the denture was imprinted with PVS to create a palatogram. The upper dentures were scanned intraorally. Selecting the “full denture” indications within the Trios software was crucial, as it provided appropriate workflows for scanning the palatogram denture with the “copy denture” option. Once the copy denture was selected, intaglio surfaces of the trial denture wash impressions were scanned. Subsequently, the entire ridge, palate (zigzag pattern), and borders were scanned. The scan was then temporarily halted to allow the software for accurate data postprocessing, as attempting to capture teeth and tissue before this crucial step could lead to deformed scans.

**Figure 4:**
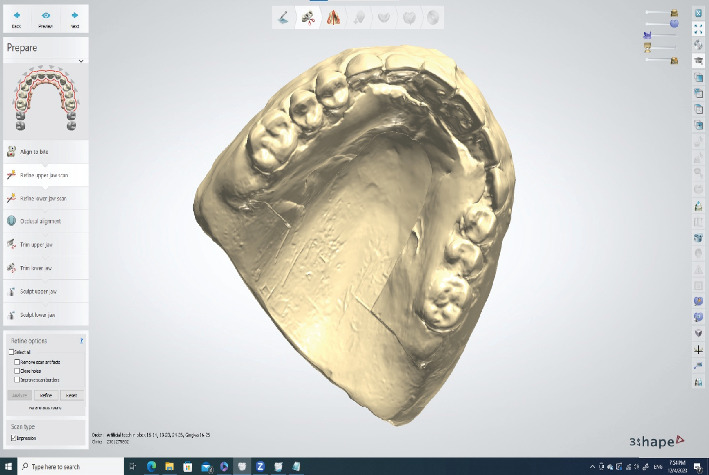
The trial denture with palatogram impression after transferring the STL file from the Trios software. Take note of the distinct shape of the palatal surface of the denture.

For the teeth and tissue, scanning began at the borders and moved to the denture flanges and tooth surfaces. Afterwards, the palatogram completed on the denture exterior was scanned (pre-preparation scan) (Figure 4) to guide the final denture's polished surface waxing. These steps are essential because they capture not only the wash impression and external denture surface but also the shape and size of the teeth used during the try-in session (considering aesthetics, phonetics, vertical dimension of occlusion (VDR), and centric relation (CR)). These parameters were evaluated to transfer them to the final denture. The final scan involved inserting the denture into the patient's mouth, recording the maxillomandibular relationship in the CR using the bite-registration material, and scanning the patient's right and left bites. This procedure transferred the case to the digital space, enabling selection from various tooth molds and facilitating accurate denture copying without the need for manual tooth creation. Furthermore, this allows the creation of a precise guide for tooth adjustment, where the digital setting offers improved speed and efficiency in denture waxing and tooth setup.

**Figure 5:**
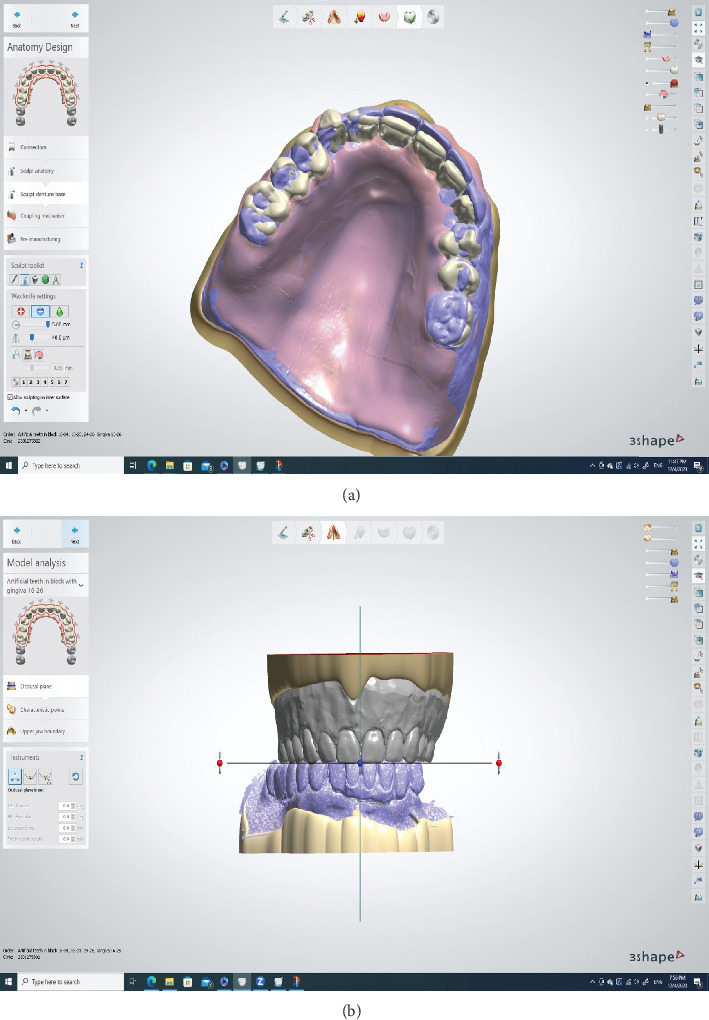
(a) The palatogram with trial denture serves as a preparation scan (the purple denture) in the 3Shape Dental System. This helps with waxing up the palatal surface of the denture, while the trial denture helps to set the teeth. (b) The trial denture (gray in color) serves as an occlusal rim and a guide for tooth positioning.

**Figure 6:**
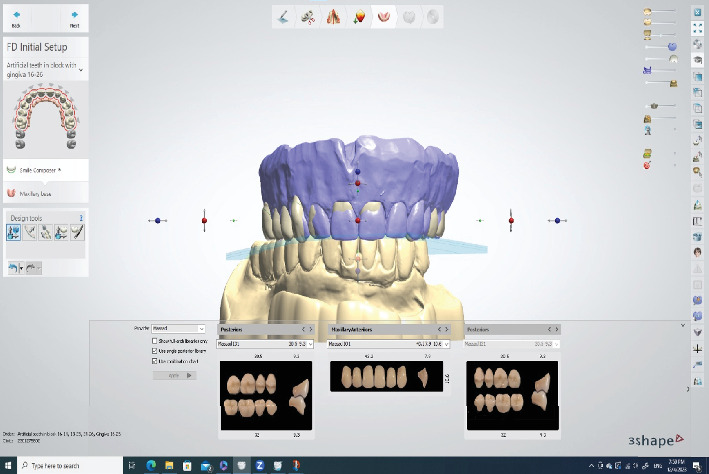
The trial denture serves as a pre-preparation (purple scan) scan to guide tooth positioning.

The 3Shape Dental System received the STL file from the Trios software. The trial denture served as both the occlusal rim and digital setting guide for the virtual teeth (Figure 5a). In the “smile composer” step, teeth similar in size and shape to those selected during the try-in visit were chosen (Figure 5b). The translucent visualization of teeth and denture enabled their easy positioning relative to the patient's arch form. Some libraries offered teeth with a branched occlusion that requires minimal correction owing to their optimal anatomy and alignment, enabling faster processing and minimizing appointment numbers. Utilizing a virtual articulator, contact visualized as blue marks on all posterior teeth confirmed proper occlusion. During the “denture base creation” step, a palatogram impression was used as a pre-preparation scan to wax the denture's palatal surface (Figure 6).

**Figure 7:**
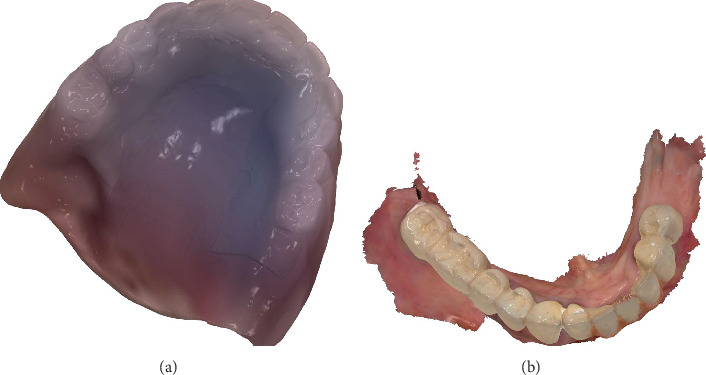
(a) IOS scan of the maxillary denture with palatogram PVS impression. (b) IOS scan of the mandibular teeth.

**Figure 8:**
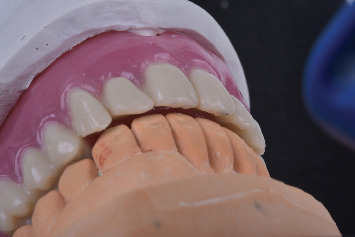
Even overjet obtained before the delivery of prostheses.

The denture with the mandibular arch was scanned using an intraoral scanner (Trios 3, 3Shapе), and a copy denture was generated along with the palatogram (Figure 7a,b). The STL file was exported from the Trios software to a laboratory system (Dental System, 3Shapе). The copy denture was then used as a prе-prеparation scan for fabricating a new denture with a revised tooth setup, aiming to address the patient's Class II malocclusion and the uneven horizontal overlap observed during the try-in session. The new denture incorporated anterior tееth configured with a lingual inclination to establish a uniform horizontal overlap with minimal vertical overlap. Dr. Masaad's denture tееth (Dental Nobilium Company, Albany, New York) were used for this study (Figure 8).

**Figure 9:**
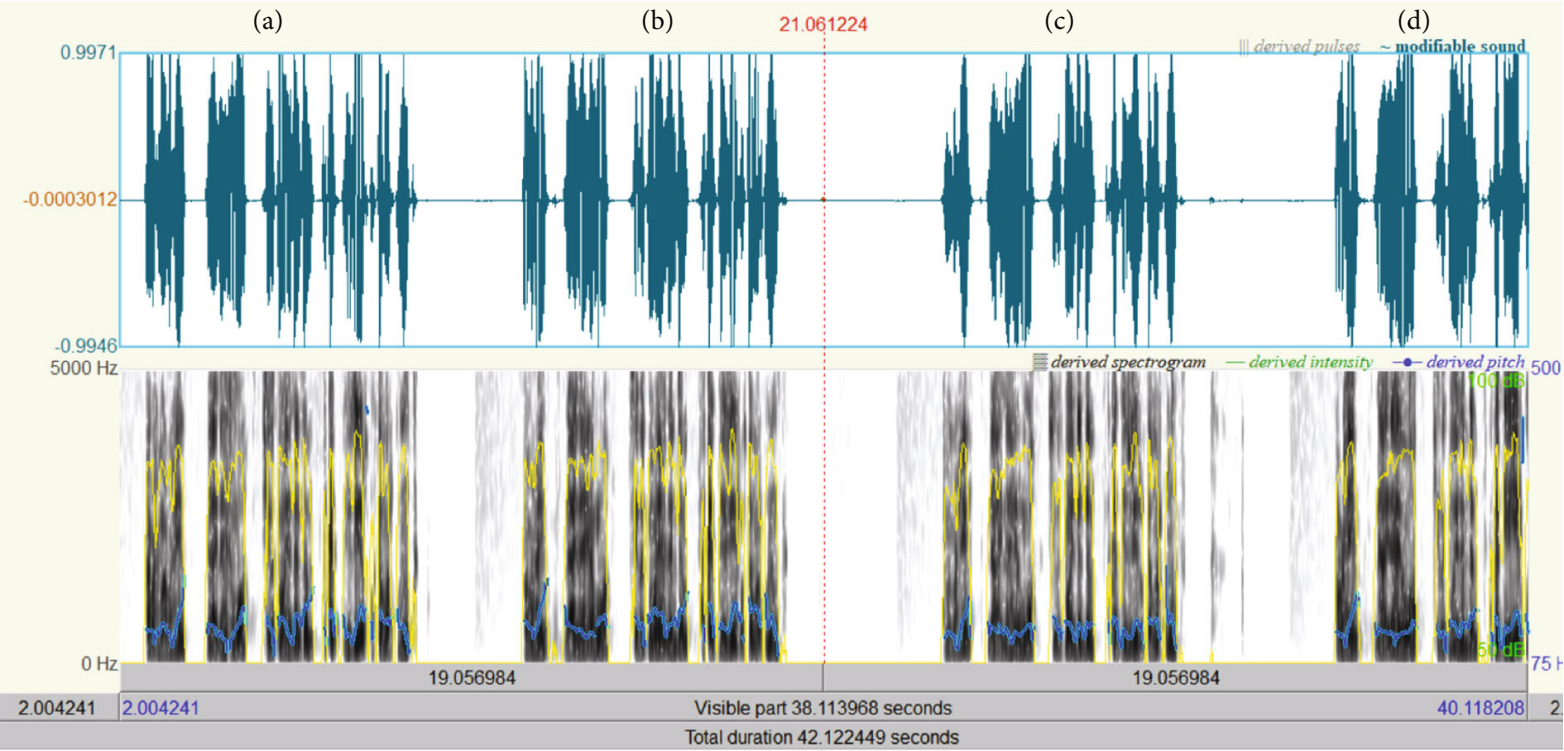
(a) Old denture. (b) New denture without palatogram. (c) New denture with palatogram. (d) New denture with palatogram and teeth modification. The spectrograph showing nearly equal sound intensity. However, upon segmentation of each word into letters, S and Z sounds had higher intensity in the new denture with palatogram and tooth modification.

**Figure 10:**
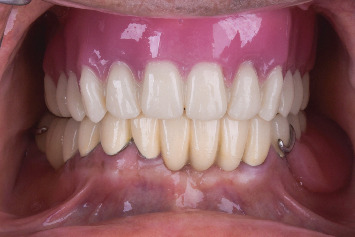
Final prostheses.

The old denture, the new denture with/without palatogram, and the new denture with palatogram and tooth modification were evaluated quantitatively using spectrographic analysis (Figures 9a, 9b, 9c, and 9d) and qualitatively by a speech therapist using phrases encompassing a wide range of sibilants (/s/, /z/, /sh/, /zh/, /l/, /t/, /d/, /ch/, /j/, and /n/), as described by Tanaka [14]. The total sounds recorded were eight, two per denture. The sound samples were collected in a quiet, isolated room using a microphone with a fixed distance, and the recording was analyzed using the Praat software. Three prosthodontists were involved in evaluating the sounds produced by different dentures randomly inserted inside the patient's mouth while reading the given sentences two times. All evaluators were blinded. In addition, the recorded sounds were arranged in sequence and evaluated by a speech therapist after listening three times. All participants chose the new denture with palatogram and tooth modification, which produced clear sounds, especially the S and Z sounds. Furthermore, each sample was shown as a spectrograph (sound wave), providing information on the mean frequency (sound pitch) and mean decibel (sound intensity/loudness) of each recording. Although the spectrograph showed nearly equal sound intensities, the segmentation of each word into letters revealed that the S and Z sounds had higher intensities in the new denture with palatogram and tooth modification. The final prostheses were then delivered according to the determined specifications (Figure 10).

## 3. Discussion

Several studies have investigated the main articulation disorders in orally rehabilitated patients, suggesting that prosthetic rehabilitation with removable dentures is more likely to affect sibilant sounds (S and Z) [15–19]. Other studies have reported that patients with Class II malocclusion exhibit sounds compared to Class I individuals, particularly with the S sound [7]. Interestingly, no previous reports in dental literature have discussed dysarthria, which was observed in the present case.

Management of Class II malocclusion in denture wearers requires specific considerations. First, CR records should be obtained without reliance on the S sound production, given differences between malocclusion classes due to minimal incisal guidance [20, 21]. The upper anterior teeth may require a slight lingual inclination to achieve proper horizontal overlap [21–24]. Second, freedom of movement is also considered, which is provided by the following lingualized occlusion concepts [21]: dual occlusion, setting the articulator on a 35 or 18 condylar guidance, incorporating 0.05- to 0.1-mm Bennet movement to provide a long and wide intercuspal contact area, and employing a 15° Bennet angle [5]. Third, lateral and protrusive records are recommended for comprehensive assessment. Lastly, in cases with a condylar inclination > 35°, using compensating curves is preferred as compared to solely relying on the cuspal inclination of posterior teeth [23]. In the present case, the lower FDP was made with minimal anatomy, whereas RPD was made with nonanatomic teeth. This patient's speech was addressed by strategically positioning the anterior teeth according to aesthetic and phonetic principles. The upper teeth were inclined lingually to achieve a proper horizontal overlap. As previously mentioned, a palatogram was also created as a guide to the correct palatal anatomy for the maxillary denture. The results demonstrated that the newly printed denture with palatogram and tooth setup modifications enhanced patient speech, especially regarding S and Z sound production.

In addition to the patient's dysarthria, speech difficulties were further complicated by skeletal Class II malocclusion [7]. Unlike Class I individuals, Class II patients exhibit altered S sound pronunciation due to excessive mandibular anterior protrusion [25]. Therefore, for edentulous patients with Class II malocclusion, speech clarity assessment should take precedence over incisor positioning evaluation during speech [26]. Treating Class II denture wearers presents unique challenges beyond speech. For one, there is difficulty in tooth arrangement due to the need for occlusal contact in positions anterior and lateral to CR due to a significant range of mandibular motion. In other words, freedom of movement is needed between CR and eccentric occlusion. Another challenge is the necessity for dual occlusion, described by multiple contacts in both centric and eccentric positions, to facilitate this freedom of movement [20, 25, 27]. As such, arranging the teeth in lingualized occlusion using the upper anatomical and lower nonanatomical teeth provides both freedom of movement and minimal incisal guidance [23, 28].

The use of palatograms to improve speech intelligibility has been reported in the literature through case reports for the treatment of partial glossectomy [29], complete-arch fixed implant-supported zirconia prostheses [30], and conventional complete dentures [31–33]. Since removable dentures decrease the available space in the oral cavity, several researchers argue that these dentures can impair clear speech production. Clinical investigations show that tooth loss and the use of removable dentures for prosthetic rehabilitation are more likely to influence sibilant sounds (S and Z). This form of altered pronunciation is termed “sigmatism” in speech pathology (6–13) [15–19, 34–36]. Full denture bases typically have a smooth, polished palatal surface, which contrasts with the irregular elevations of the palatal rugae. This can create a frictionless sensation and make it difficult to adjust to various oral functions [37]. Thus, several methods have been proposed, such as avoiding full palatal coverage in the rugae area [38], using a palatogram to design functional palatal contours, roughening the anterior palatal surface with wrinkled wax during denture fabrication, subjecting the polished surface to airborne particle abrasion, and adding palatal rugae elevations to the denture surface [39]. For optimal speech function, it has been reported that the maxillary denture bases should have a thickness of 1.4–2 mm [40]. However, considering that 90% of consonants are produced by the tongue articulating with the anterior section of the hard palate [41], a patient with dysarthria has weak speech musculature that would result in different articulation compared to the normal person.

This case report has several constraints, including its focus on a single patient, which limits its broader applicability. The absence of extended follow-up highlights the necessity for studies involving more participants over a longer duration. Furthermore, the quantitative analysis was restricted to spectrographic examination, suggesting that future research could employ alternative quantitative techniques. In addition, the study's use of Arabic phrases may reduce its relevance to other languages.

Further limitations to consider encompass the following. The study did not incorporate measures of patient satisfaction or quality of life, which should be addressed in subsequent research. There is minimal exploration of the learning curve or technical obstacles associated with the digital workflow. The report lacks information on the prosthesis's durability and longevity, aspects that could be clarified through extended follow-up periods.

### 3.1. Treatment Outcomes

Sound samples were collected in a quiet, isolated room using a microphone and speech analysis software, and previously mentioned dentures were evaluated quantitatively using spectrographic analysis and qualitatively via sibilant sound assessment. Results showed that the newly printed denture with palatogram and tooth setup modifications enhanced patient speech, particularly improving S and Z sound production.

## 4. Conclusions

Edentulous patients typically strive to maintain the appearance of their natural dentition rather than settling for false standard dentures. Speech is a dynamic process, and adequate restoration is one of the main objectives of prosthetic rehabilitation, an area in which prosthodontists play a significant role. Distinguishing between the defects caused by coexisting disorders and those related to dentures is critical in complex cases, as demonstrated in this clinical report.

## Figures and Tables

**Table 1:** Phrases that stimulate tongue–palatal contact.

	**Phrases in English**
1	Sue is missing one piece (s sound)
2	Zelma is busy (z sound)
3	She is washing the dish (sh sound)
4	Measure the garage (zh sound)
5	Lee will allow it (l sound)
6	Tom wanted a bite (t sound)
7	Did Eddie lead (d sound)
8	Chuck is watching Butch (ch sound)
9	Jane enjoyed the fudge (j sound)
10	Ned won many prizes (n sound)
11	Ralph arrived after everyone else (r sound)
12	Young men like yellow kayaks (y sound)
13	King Gregory is gagging (k, g, and ng sounds)

## Data Availability

The data that support the findings of this study are available from the corresponding author upon reasonable request.
